# The Institutional Worker

**Published:** 1913-07-12

**Authors:** 


### INSTITUTIONAL WORKER

**Being a Special Supplement to "The Hospital"**

### OUR BUREAU OF INFORMATION.

### Rules for Correspondents.

1. Every letter must be accompanied by the coupon to be cut from the back cover (inside page) of The Hospital, current issue, and must contain the name and address of the correspondent with pseudonym for publication if desired. Replies by post cannot be given, save under exceptional circumstances at the Editor's discretion.

2. Letters from Approved Homes in reply to special needs published in the Bureau should state terms and full particulars, and be sent prepaid under cover to the Editor of the Bureau with name written across coupon for identification.

3. Proprietors of Homes which have not yet been entered on the List of Approved Homes, but have spare accommodation likely to suit special needs, are invited to write for an application form for registration. The fee for registration, which includes two announcements of the Home in the Bureau and other privileges, is 10s.

4. All communications to be addressed to the Editor of The Hospital, 28 Southampton Street, Strand, London, W.C., and marked "Bureau of Information."

### *THE INSTITUTIONAL OFFICER.*

Contributions from officers engaged in the work of institutions for the relief of the sick and injured, whether voluntary or rate supported, are invited, and will be paid for in accordance with scale if made use of.

### An Opportunity for Mutual Help.

The paper read by Mr. Keith D. Young at the Conference of the British Hospitals Association was full of practical suggestion bearing upon the construction and maintenance of hospital buildings, and aroused an enthusiastic and instructive debate. The many points of technical detail touched upon should, however, be fruitful of a much fuller exchange of experiences than was possible at the Conference, for the discussion was necessarily limited to comparatively few representatives of the hospitals. These columns offer an opportunity to all hospital workers to continue the discussion week by week. There are many experienced officers who could, we are confident, illuminate the numerous problems involved in questions of technical detail, the methods of handling which have such an important effect upon efficiency and economy, and we invite our readers to make use of these columns as a platform for the dissemination of their experience and knowledge.

### *THE SICK AND IN NEED.*

**The Editor is prepared to make known without charge the needs of any who may be sick or in difficulties, and to guide them in making choice of Homes for treatment or convalescence. Reduced terms can often be arranged with Homes on the Approved List for those unable to pay full fees. Queries are specially invited from those who are engaged in any kind of philanthropical work. A new edition of the leaflet describing the purposes of the Bureau of Information is now ready and will be sent free of charge on application.**

### Help Required for a Family in Need.

This is a very sad case and merits the fullest sympathy and generosity of our readers. As stated in our last week's issue the family is in deep distress. The father contracted phthisis some time ago, and the progress of the disease has compelled him, after repeated breakdowns, to relinquish his business career. He has a wife and two little children dependent upon him; his private means are exhausted, and his income is limited to the sickness benefit of 10s. weekly to which he is entitled under the National Insurance Act, and a small allowance in kind, in lieu of sanatorium treatment which is not forthcoming. The mother prior to her marriage was an elementary school teacher, and she could, were she able to get work, support herself and her children by teaching. Meanwhile the family is in urgent need of help. There are several ways in which this might be rendered, as, for example, the provision of monetary assistance to tide over the present crisis, the reception of the father into a suitable Home where he could be cared for free of charge, or the employment of the mother in some suitable capacity whereby she may be enabled to support herself and her children. Co-operation can accomplish so much in a matter of this sort, and such help as our readers can give in one or other of the directions indicated will be gladly welcomed. The family is at present residing in the neighbourhood of Folkestone. Replies should be addressed "M. E. F.," 167b.

### Prevention of Consumption.

The National Association for the Prevention of Consumption publishes a list of sanatoria in the country with full particulars as to admission, which you will find helpful. This Association had its inception about thirteen years ago, and is actively engaged in the education of public opinion upon the question of the prevention of tuberculosis. Its system of propaganda is comprehensive and well organised. It arranges for public lectures by approved lecturers, and generally by the collection and distribution of data as to modes of diffusion and measures of prevention of tuberculosis acts as a bureau of information. The Association also co-operates with other societies having for their object the promotion of public health, and assists in the establishment of sanatoria. Local branches have been established throughout the kingdom, and the Association has brought itself into close co-operation with similar agencies in the Colonies, and indeed throughout the world. The passing of the National Insurance Act has opened up a new field for its activities, and the past year has consequently witnessed many developments in its educational policy. Particulars as to membership may be obtained of the Secretary, 20 Hanover Square, London, W.—"Interested."

### *INSTITUTIONAL FACTS AND FIGURES.*

#### QUESTIONS FOR JULY.

**No. 1. (For the Officers of all Medical Institutions.)—State the character of your Institution, and describe your system of recording the admission, treatment, and discharge of patients admitted to the wards. Give descriptions of the various forms in use for the purpose, the method of dealing with them in the process of registration, and the method of filing history and treatment sheets. Add your further remarks.**

**No. 2. (For the Officers of Voluntary Hospitals.)—If you keep your accounts in accordance with the Uniform System, describe fully your method of checking, sorting, analysing, and filing your accounts for payment. Give such particulars as will clearly indicate the manner in which these are passed through your analysis journal, and onwards through the ledger. Add your further remarks.**

#### RULES.

The following rules must be observed:—

1. Contributions must be written on one side of the paper. They must bear the name and address of the sender and be accompanied by coupon to be cut from the back cover (inside page) of the current issue of The Hospital. A pseudonym must be chosen if the name is not to be published.

2. They must be addressed to the Editor of The Hospital, 28 & 29 Southampton Street, Strand, London, W.C., so as to reach him before the end of the month, and must be marked in the left-hand corner "Facts and Figures."

**A minimum payment of Half a Guinea will be made for each published answer.**

### *THE INSTITUTIONAL ARTIFICER.*

### Bed Truck.

The inquiry under this heading will be dealt with next week.—" Hospital Secretary."

### To Prevent Beds Damaging Walls.

How to do this is a question which interests everybody, because in no two institutions are the devices the same. Fixing rubber pads or buffers to the back of the beds has been tried with moderate success, and a useful detachable buffer has recently been placed on the market. Most devices, however, for preventing the beds from touching the walls involve an obstruction on the floor which is apt to prove a dust-trap. One of the best ways of overcoming this objection is boldly to sacrifice the rounded corner where wall and floor meet, and to achieve a similar result by making their juncture convex instead of concave. In other words, fill up the corner and make the surface rounded. As regards materials, concrete on expanded metal has been tried and found satisfactory in at least one institution that we could name. If you decide to experiment with this device we should be very glad to publish your experience through the "Bureau."—" Clerk of Works."

### *PRACTICAL MATTERS.*

### Ward Fireplaces.

The choice of a ward-heating stove requires careful consideration, for not only is it necessary to select a stove which will radiate a maximum of heat with a minimum consumption of coal, but it must also be borne in mind that the efficiency of ventilation is largely dependent upon the class of stove adopted. There are many different patterns on the market which are worthy of note, but, in particular, one which seems to combine all essential qualities is the "Manchester" stove, patented by Messrs. Shorland and Brother. Experience indicates that this stove is highly efficient in maintaining an equal temperature with a relatively low fuel consumption, and what is equally important is the distribution in the ward of a large supply of pure warm air effected by its design. It is constructed with either an ascending or descending smoke flue; the latter is, however, most generally favoured, for it obviates the need for a smoke shaft which obstructs the view of the ward.—" Secretary."

### Ward Lighting.

The method of indirect lighting to which you refer is that of the British Thomson-Houston Company, known as the "Eye-Rest" system. The softness and agreeable daylight quality of indirect lighting especially commends its employment in the general lighting of hospital wards, and the system is well worth your consideration. The fittings comprise suspended bowls containing powerful reflectors; these are upturned, and the light from the lamps is first reflected to the ceiling, and thence distributed uniformly over the room. The scientific construction of the reflector, and the use of the "Mazda" drawn-wire incandescent lamp, combine in producing a high standard of efficiency. "Eye-Rest" fittings can be fixed in the place of existing ceiling fittings; they do not require any special fixing, and are connected up in precisely the same way as ordinary electric-light fittings. It may be found necessary to alter the spacing of the ceiling points, but this is neither a difficult nor expensive matter. The system has recently been introduced into the Chiswick General Hospital, and an instructive article dealing with the installation appeared in The Hospital on May 3. The only objection to the fittings which has been brought to our notice is that they collect dust and dirt, but this can scarcely be regarded as a valid criticism, for all fittings must be periodically cleaned, and the additional labour involved in attending to these is not worth mentioning.—" Matron."

### Cost of Laundry Materials.

We could better advise you as to the quantities of washing materials required in your laundry if you stated the average number of pieces washed weekly; it is possible to form only a rough estimate from the figures you give, as the allowances and requirements vary in different institutions. The cost of soap and soda, or soda substitute, averages about 5s. 6d. per 1,000 articles in the smaller hospitals. The question is of considerable interest and importance, and we shall be pleased to enter into fuller details upon the receipt of the information asked for. We should be glad to have a description of your laundry arrangements and machinery in addition. Some exhaustive articles dealing with the cost and working of certain Metropolitan and Provincial laundries appeared in The Hospital of November 16, 1912.—" Litty."

### Potato Machines.

There are several excellent potato-peeling machines on the market, the cheapest probably being those by Mabbott and Co., of Poland Street, Manchester. They are made in several sizes, the smallest of which costs £3, and will peel about 5 lb. a minute. These machines are well made and work very satisfactorily. Some well designed machines are made by the Avamore Engineering Co., Ltd.; amongst them is the "Cornhill," the price of which is £26 10s.—" Kitchen."

### EDITOR'S LETTER-BOX.

"*Wayne.*"—Thank you for your letter; we have forwarded a communication from one of our Approved Homes, and shall be glad to hear that you have been able to make a satisfactory arrangement.

*Communications and enclosures have been received from the following and duly attended to* :—M. J. Rumball and M. Greenwood.

### *THE INSTITUTIONAL HOUSEKEEPER.*

### Floor Polish.

You can considerably reduce your expenditure upon the polishing of floors if you prepare your own polish instead of using the ready-made preparations on the market. You will find the following formula useful: dissolve 28 lbs. of beeswax in 5 gallons of turpentine by gently heating; this can most safely be done by placing it in the steam steriliser. Instead of turpentine a turpentine substitute may for cheapness be used, and it answers equally well.—" Housekeeper."

### Liquid Sanitary Soap.

The liquid sanitary soap to which you refer is probably "Carbolacene," prepared by Messrs. W. and F. Walker, Ltd., of Water Street, Liverpool. It is claimed that it cleansee and disinfects clothes, carpets, blankets, etc., and can be used for washing clothes without the addition of soap for the combined purpose of cleaning and disinfecting them at the same time. The substance is easily dissolved in water, and the usual strength is 2½ per cent., or, roughly, a cupful of "Carbolacene" to a bucketful of water. It is sold in drums of five gallons and upwards.—" Sanitary."

### *EMPLOYMENT AND TRAINING.*

### Midwifery Training.

You do not say whether you have any preference as to locality. At the Liverpool Maternity Hospital, Brownlow Hill, pupil midwives are received for £15 15s. In London, among very many excellent Training Schools, are the following: The Maternity Charity and District Nurses' Home, Plaistow, E.; the City of London Lying-in Hospital, City Road, E.C.; and Queen Charlotte's Lying-in Hospital, Marylebone Road, N.W. The fees at these Institutions average from £25 to £30. The fee for the C.M.B. examination is £1 1s. There are a large number of recognised schools and teachers throughout the country, and lists of these may be obtained upon application to the Central Midwives' Board at their offices, Caxton House, Westminster, S.W. You will find "A Complete Handbook of Midwifery," by J. H. Watson, a useful book; it is published by the Scientific Press, Ltd., and the price is 6s.—" A."

### Male Nursing.

With the exception of a few men trained by the Middlesex and St. Bartholomew's Hospitals for their own services, the only Civil Hospital which trains male nurses is the National Hospital for the Paralysed and Epileptic, Queen's Square, Bloomsbury. You can get no better training than in the Army Hospitals; this you can obtain by enlisting in the Royal Army Medical Corps. Candidates must be between eighteen and twenty-five years of age.—"S.G."

### Editor's Notices.

**Contributions:**

Contributions should be written, or preferably typed, on one side of the paper only, and all articles sent in are accepted upon the distinct understanding that they are forwarded to The Hospital only.

The Editor cannot undertake to return MSS. not used, but when a stamped directed envelope is enclosed the MSS. may be returned if a special request is made.

Accepted articles and paragraphs of news will be paid for after publication at the scale rate.

**Address:**

To prevent delay all contributions and letters on editorial business must be addressed exclusively to the Editor, "The Hospital," 29 Southampton Street, Strand, London, W.C. It is important that this regulation shall be strictly observed.

**Correspondence:**

Correspondence on all subjects is invited. The name and address of correspondents must be given as a guarantee of good faith, but not necessarily for publication.

**Special Articles:**

Special articles are invited, and questions, inquiries, and paragraphs upon all matters relating to the work, administration and management of general, special, mental, fever, cottage and convalescent hospitals, sanatoria, homes, institutions, societies and organisations for the treatment or care of the sick, injured and dependants of all classes. Every matter affecting the interests and welfare of the staff of all grades working in these institutions will receive special consideration.

**Administrative Medicine, Research and Items of News:**

Special terms will be paid for approved articles on subjects in this wide field by experts who have made some section of it their special study and interest. We wish to give prominence to every movement tending to advance accuracy of diagnosis, efficiency in treatment, and the development of modern methods to eradicate disease and promote the welfare of the suffering.

**A Bureau of Information:**

We invite inquiries and applications for information and help in providing for that numerous class of sufferers who require special care or house-room which they cannot obtain in their own homes or secure for themselves. We cannot, however, prescribe, or recommend practitioners.

**Books for Review:**

Publishers are particularly requested to send advance proofs of any new books of importance whenever possible, as the Editor has made arrangements to publish immediate reviews on a new plan.

**Photographs, Plans, Blocks, and Illustrations:**

It is requested that wherever possible MSS. may be accompanied by illustrations in any of the above forms. The name of the sender and of the article to which it belongs should be written on the back of each photograph, drawing, or block for purposes of identification.

**Local Papers:**

Newspapers containing reports or news paragraphs should be marked and addressed to the Sub-Editor.

**The Coupon System:**

A coupon will be found at the bottom of the third inside page of the cover each week. This coupon must be attached to every question or inquiry to which an answer is desired in The Hospital.

### The Workers' Help.

Our purpose is to make this Supplement of The Hospital of the greatest possible service to every Institutional Worker. The term is employed in its widest sense, and includes all those individuals engaged in or associated with Charitable, Poor-Law, and Municipal Administration, as well as the Medical, Health, and Sanitary Services.

Although the section is primarily intended to help those seeking appointments, and numerous announcements of vacancies appear in its columns every week, it is felt there are also many other ways in which it can be of value. A number of pages have recently been added containing information which it is hoped will prove of assistance to many of our readers in their work, and in this you can help us and yourself by showing a lively interest in this section and offering such suggestions for its improvement as may occur to you.

### Manager's Notices.

**Letters relating to the publication, sale, and advertising departments of "The Hospital" must be addressed to The Manager, "The Hospital," The Hospital Building, 28 and 29 Southampton Street, London, W.C.**

**Advertisements:**

To ensure insertion, all advertisements for The Hospital must reach the Manager not later than Wednesday morning in each week. For scale of charges see pages v and 4.

**Special Rates** will be quoted for those institutions that agree to send all their vacancy, contract, and public notices to The Hospital each year, so as to make it a file of such announcements for ready reference.

**Subscriptions** may begin at any time, and are payable in advance. Cheques and Post-Office Orders should be crossed London County and Westminster Bank, Covent Garden Branch, and made payable to the Manager of The Hospital.

Rates of Subscription (including postage).

| *United Kingdom.* | | *Foreign and Colonial.* | |
|---|---|---|---|
| Three Months ... | ... 2s. 0d. | Three Months ... | ... 2s. 6d. |
| Six Months | ... 4s. 0d. | Six Months ... | ... 5s. 0d. |
| Twelve Months ... | ... 6s. 6d | Twelve Months ... | ... 8s. 8d. |

*SUBSCRIPTION FORM.*

*Please send me* The Hospital *for..............months, commencing with your issue of.............................., for which please find enclosed.............................*

*Name*.........................................................

*Address* .........................................................

.........................................................

*Date*.......................................

*To* ....................................... *Newsagent.*

**Remittances:**

**It is especially requested that remittances be made by Cheque or Postal Order, and in halfpenny stamps only when the amount is under sixpence.**

NEARLY

**400 NEW WORDS**

have been added to the

**NEW EDITION**

of

### The Nurse's Pronouncing Dictionary

**OF MEDICAL TERMS AND NURSING TREATMENT**

Compiled by **HONNOR MORTEN.**

Eighth Edition, thoroughly Revised and much Enlarged.

Edited by **MARY I. BURDETT.**

The new issue of this well-known Dictionary is the **most complete work** of the kind published. It has been completely revised and some hundreds of new words have been added, consequently the utmost reliance can be placed in the information it contains.

To be up-to-date you should GET A COPY TO-DAY.

**Price, in cloth, 2/- post free; in leather, 2/6 net, by post 2/8.**

*Of all Booksellers, or of—*

**The Scientific Press, Ld.**

(*The Nurses' Publishers*),

**28/29 Southampton Street, STRAND, LONDON, W.C.**

### HANDBOOKS
### For TRAINED WORKERS

POCKET-GUIDE SP SERIES

**1/- net. 1/1 Post free.**

**Bound in Limp Cloth.**
**Suitable Size for the Pocket.**

**The works which comprise the S.P. Pocket Guide Series are intended for ready reference, consequently the aim of the Publishers has been to make them as concise and lucid as possible. Each book is written by an expert on the subject of which it treats, and the utmost reliance therefore can be placed in the information it imparts.**

**Essentials of Fever Nursing.** By LYTTON MAITLAND, M.D. (Lond.), M.B., B.S., D.P.H. (Camb.).

**Manual and Atlas of Swedish Exercises.** (*With over 60 Illustrations.*) By THOMAS D. LUKE, M.D., F.R.C.S.

**Bandaging Made Easy.** (*Illustrated with over 90 Diagrams.*) By M. HOSKING, Sister-in-Charge, Tredegar House, Bow, E.

**How to Write and Read Prescriptions.** By LYTTON MAITLAND, M.D. (Lond.), M.B., B.S., D.P.H. (Camb.).

**Principal Drugs and their Uses.** By A PHARMACIST.

**Asepsis and How to Secure It.** By H. W. CARSON, F.R.C.S.

**Treatment after Operations.** *Rules for Nursing after General and Special Operations.* By MARY WILES.

**The Nurse's Duties before and during Operations.** By MARGARET FOX, Matron, Prince of Wales's General Hospital, Tottenham.

**Other works, in amplification of this Series, will be announced in due course.**

*Published by*

**The SCIENTIFIC PRESS, Ltd.**

**28/29 Southampton Street, STRAND, LONDON, W.C.**

